# Inhibition of Diffusion Flames by Methyl Bromide and Trifluoromethyl Bromide Applied to the Fuel and Oxygen Sides of the Reaction Zone

**DOI:** 10.6028/jres.065A.039

**Published:** 1961-08-01

**Authors:** E. C. Creitz

## Abstract

The difference in extinguishing effectiveness of an inhibitor introduced on the two sides of the reaction zone of diffusion flames has been measured as a function of oxygen concentration in the O_2_-N_2_ mixture supplied to the flames. Six fuels and two inhibitors were used. It was found that when the inhibitor was added to the fuel, the volume percentage required for extinguishment was much greater than when added to the oxygen side of the reaction zone, with the single exception of CO flames inhibited by trifluoromethyl bromide. In all cases except the latter, the amount required for extinction increased with increase of the oxygen concentration, being relatively less dependent on oxygen concentration above a certain threshold in the neighborhood of 21 percent when the inhibitor was added to the fuel. Above oxygen concentrations on the order of 25 percent, methyl bromide was completely ineffective when added to the oxygen side of the reaction zone, and above about 32 percent oxygen it was ineffective when added to the fuel, since at this oxygen concentration it burns without additional fuel.

## 1. Introduction

It was noted, in the course of some preliminary work on the inhibition of propane diffusion flames by methyl bromide, that extinguishment was more readily achieved when the inhibitor was supplied to the air side of the reaction zone than when added to the fuel. A search of the literature revealed that Simmons and Wolfhard [1]^1^ had reported this effect in a paper devoted primarily to the spectroscopy of diffusion flames. Because of the possibility, suggested by this observation, that the inhibitor interferes with some reaction involving oxygen or oxygenated intermediates, it was decided to study the effect as a function of the oxygen concentration in the oxygen-nitrogen mixture supplied to the flame. In addition, it seemed desirable to inhibit the flames with nitrogen added to the fuel so that a datum could be established for estimating the relative efficiencies of the other inhibitors under similar conditions.

## 2. Apparatus and Procedures

No attempt was made to obtain gases of exceptionally high purity, since the study was aimed primarily toward obtaining information about the differences between the fuel and oxygen sides of diffusion flames. The hydrogen used was prepared commercially by electrolysis and contained^2^ not more than 0.2 percent impurity which consisted almost entirely of oxygen and nitrogen. Mass spectrometer analysis of the natural gas used indicated that it consisted of 95.15 percent methane, 2.84 percent ethane, 0.63 percent propane, 0.62 percent CO_2_, 0.42 percent N_2_, and a number of heavier hydrocarbons totaling 0.34 percent. The following gases were of commercial purity, the percentage of the major component being specified with no indication of the nature of the minor components: ethane 95 percent, propane 99.5 percent, butane 99.5 percent, carbon monoxide 99.5 percent, methyl bromide 99.4 percent, trifluoromethyl bromide 94.0 percent.

**Figure 1:**
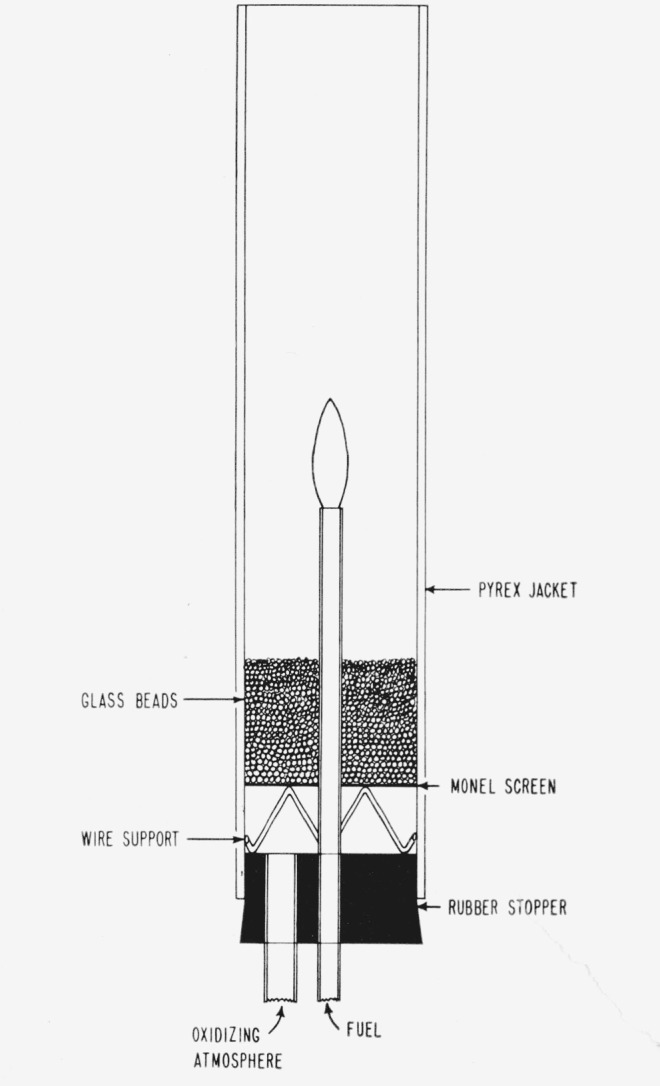
The burner used to determine the extinction characteristics of methyl bromide and trifluromethyl bromide on diffusion flames of various fuels (shown in cross section).

A sketch of the burner is shown in figure 1. The diffusion flame burned on the end of a Pyrex glass tube 7 mm o.d. with 0.8 mm wall thickness. The burner jacket was of 5 cm i.d. and had 18 cm height above the glass beads used to distribute the air flow. The height of the jacket was approximately 15 cm above the burner tube. Flow rates of the various gases were metered through calibrated flow meters. Flows of secondary air in the jacket were maintained high enough so that concentration changes caused by the flames were restricted to the immediate vicinity of the flame and back diffusion of room air into the top of the jacket was avoided.

**Figure 2:**
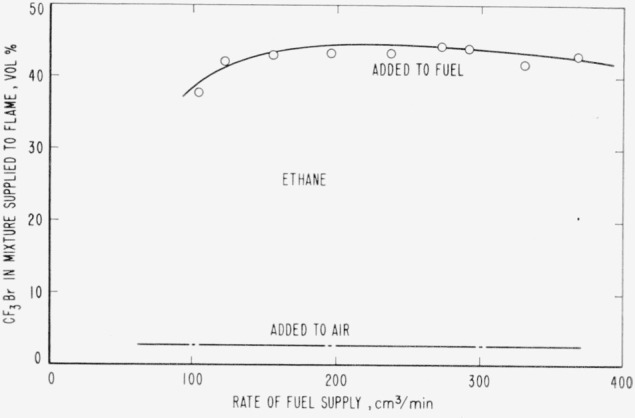
Extinction characteristics of trifluoromethyl bromide when added to ethane and when added to the air supplied to the ethane diffusion flame, as a function of the fuel rate.

Extinction of a diffusion flame may be effected by a number of factors, among them being the rate at which the fuel is supplied to the burner and the velocity of the secondary air past the flame. The latter effect was found to be relatively unimportant except at rather low or very high flow rates and was ignored. However, when the rate of fuel supply was too low, for a given burner size, the flame would not burn, and conversely, when the rate was too high, lifting occurred and the flame tended to float off and be extinguished. Between these two extremes, an optimum rate of fuel supply was determined for each of the fuels and for each of the halogenated inhibitors. A typical extinguishment curve is shown in figure 2 (the extinction region lies above the line in each curve). It can be seen that considerable deviation from the optimum value of 220 cm^3^ per minute (16 cm/sec lineal velocity) for ethane was permissible with this burner when the inhibitor was added to the fuel and the effect was very small when the inhibitor was added to the air supplied to the flame. Optimum rates for the other gases ranged between 100 and 360 cm^3^/min.

In general, a set of conditions was established and inhibitor added slowly, either to the fuel or to the mixture supplied to the oxygen side of the flame, until the flame was extinguished. Under some conditions, the flame lifted and floated as much as 10 to 15 cm above the burner where it seemed to be quite stable and would return to the burner if the concentration of inhibitor was reduced. When this occurred, a very large amount of inhibitor was required to accomplish the extinguishment. Under these conditions, the flame was called extinguished at the inhibitor concentration which caused it to suddenly pull away from the burner and rise 1 to 2 cm above it. It was felt that, in cases of very high lifting, a certain amount of oxygen was mixing with the gas as primary oxygen and the flame was no longer a true diffusion flame.

## 3. Results

**Figure 3:**
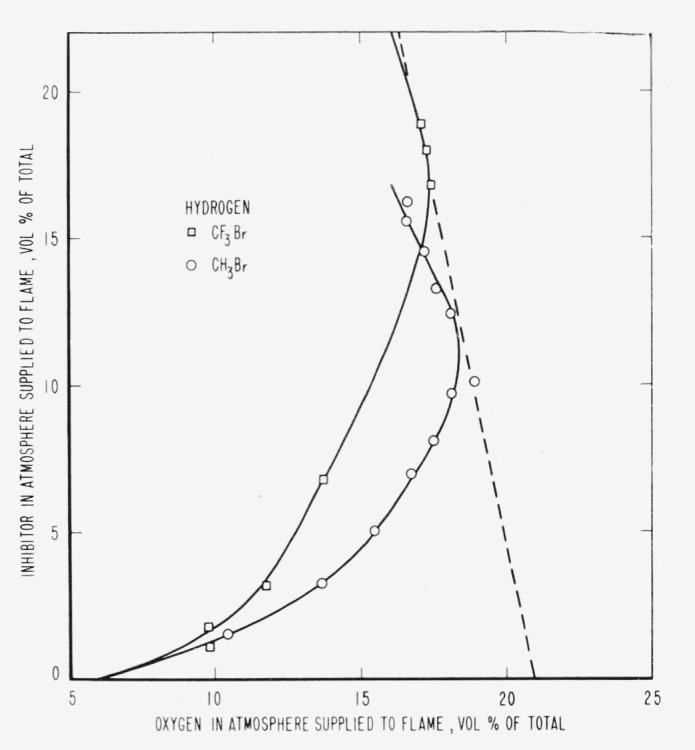
Extinction of hydrogen diffusion flames by *CF_3_Br* (□) and *CH_3_Br* (○) when the inhibitor is added to the air supplied to the flame. Flames burn in areas to the right of each curve and are extinguished to the left.

**Figure 4 f4-jresv65an4p389_a1b:**
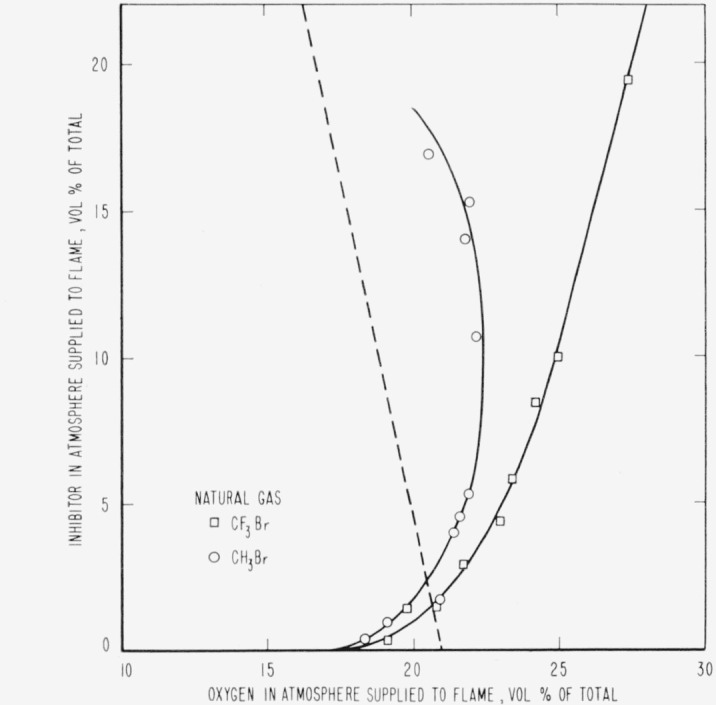
Extinction of natural gas diffusion flames by *CF_3_Br* (□) and *CH_3_Br* (○) when the inhibitor is added to the air supplied to the flame. Flames burn in areas to the right of each curve and are extinguished to the left.

**Figure 5 f5-jresv65an4p389_a1b:**
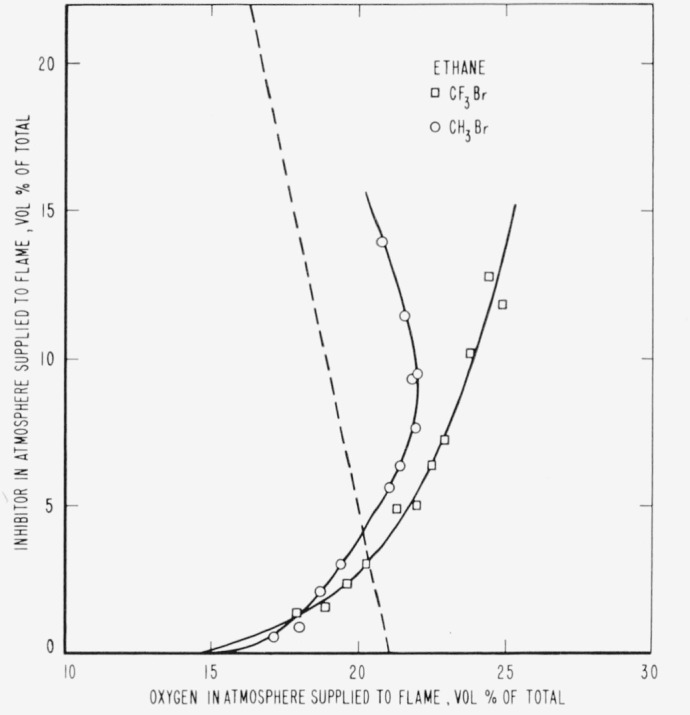
Extinction of ethane diffusion flames by *CF_3_Br* (□) and *CH_3_Br* (○) when the inhibitor is added to the air supplied to the flame. Flames burn in areas to the right of each curve and are extinguished to the left.

**Figure 6 f6-jresv65an4p389_a1b:**
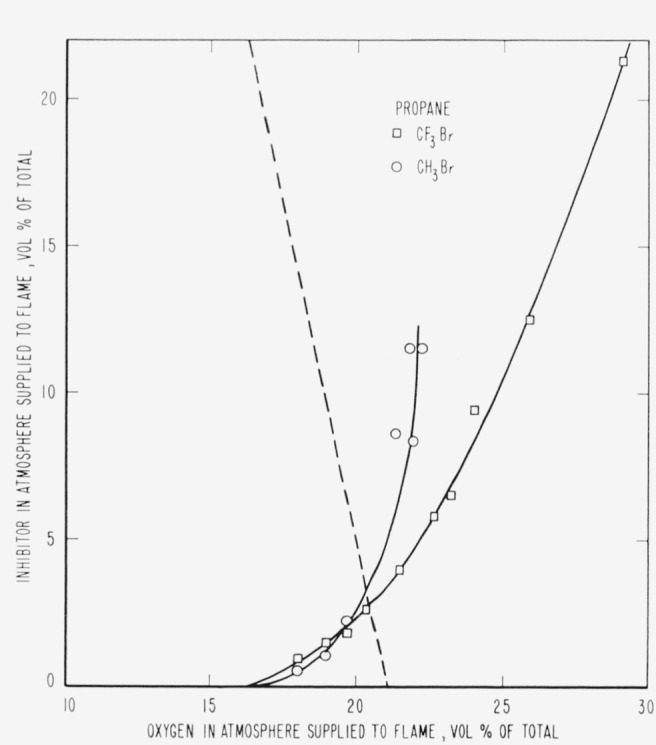
Extinction of propane diffusion flames by *CF_3_Br* (□) and *CH_3_Br* (○) when the inhibitor is added to the air supplied to the flame. Flames burn in areas to the right of each curve and are extinguished to the left.

**Figure 7 f7-jresv65an4p389_a1b:**
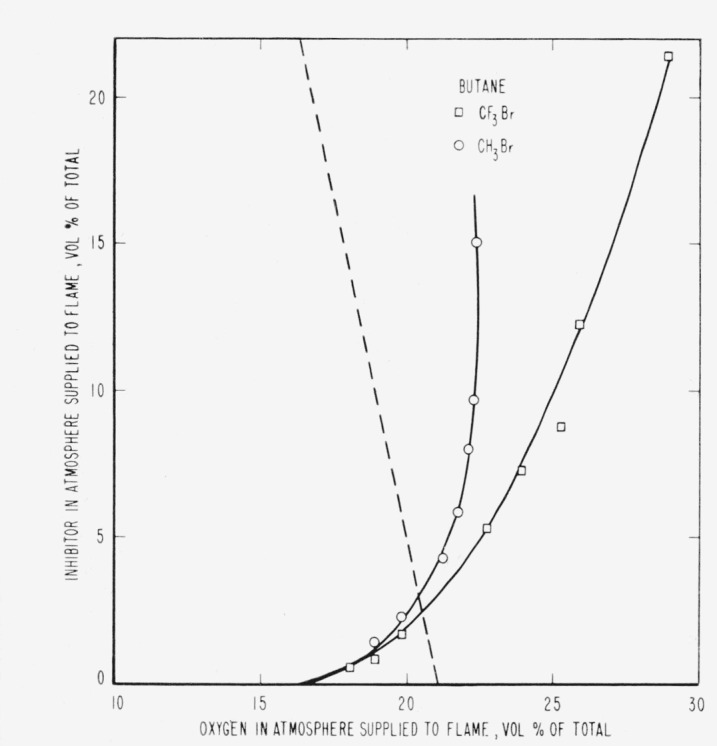
Extinction of butane diffusion flames by *CF_3_Br* (□) and *CH_3_Br* (○) when the inhibitor is added to the air supplied to the flame. Flames burn in areas to the right of each curve and are extinguished to the left.

**Figure 8:**
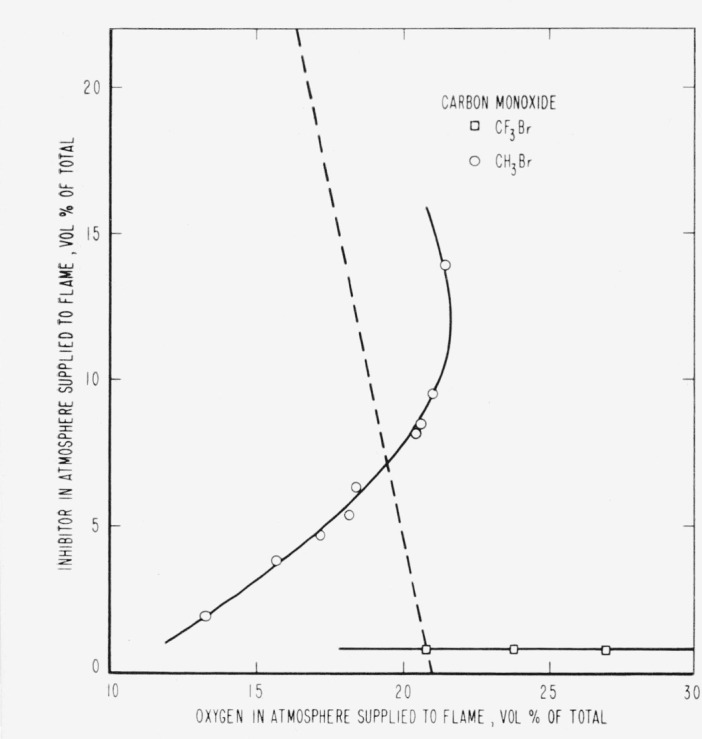
Extinction of carbon monoxide diffusion flames by *CF_3_Br* (□) and *CH_3_Br* (○) when the inhibitor is added to the air supplied to the flame. Flames burn to the right of the CH_3_Br curve and below the CF_3_Br curve and are extinguished to the left of the CH_3_Br curve and above the CF_3_Br curve.

The results are presented in the form of curves which define the boundary between burning and extinguishment conditions. Figures 3 to 8 inclusive show these regions when the inhibitor was added to the oxygen side of the reaction zone. Burning regions are to the right and below the curves while extinguishment results for conditions represented to the left and above the curves. Dotted lines represent the decrease in oxygen concentration produced by dilution of air by the inhibitor. It was impossible to obtain curves similar to those of figure 2 for hydrogen. The reason for this is shown in figure 3, which indicates that this dilution effect was important in the extinguishment of hydrogen flames in air. If the oxygen concentration had been maintained at a constant 20.94 percent independent of the addition of inhibitor, neither of the agents would have been able to extinguish the flames. [Fig f4-jresv65an4p389_a1b] [Fig f5-jresv65an4p389_a1b] [Fig f6-jresv65an4p389_a1b] [Fig f7-jresv65an4p389_a1b]

**Figure 9:**
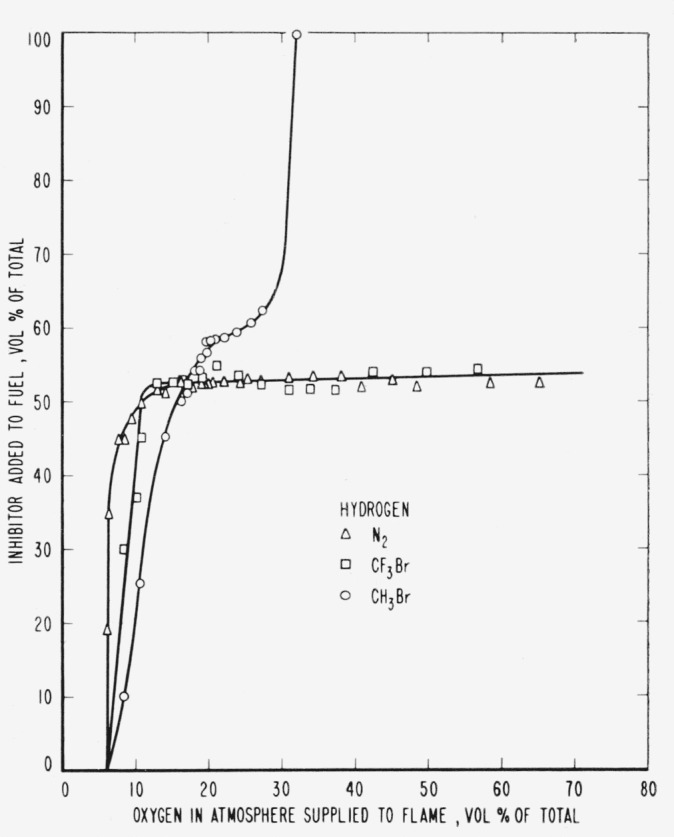
Extinction of hydrogen diffusion flames by nitrogen (Δ), *CF_3_Br* (□) and *CH_3_Br* (○) when the inhibitor is added to the fuel.

**Figure 10 f10-jresv65an4p389_a1b:**
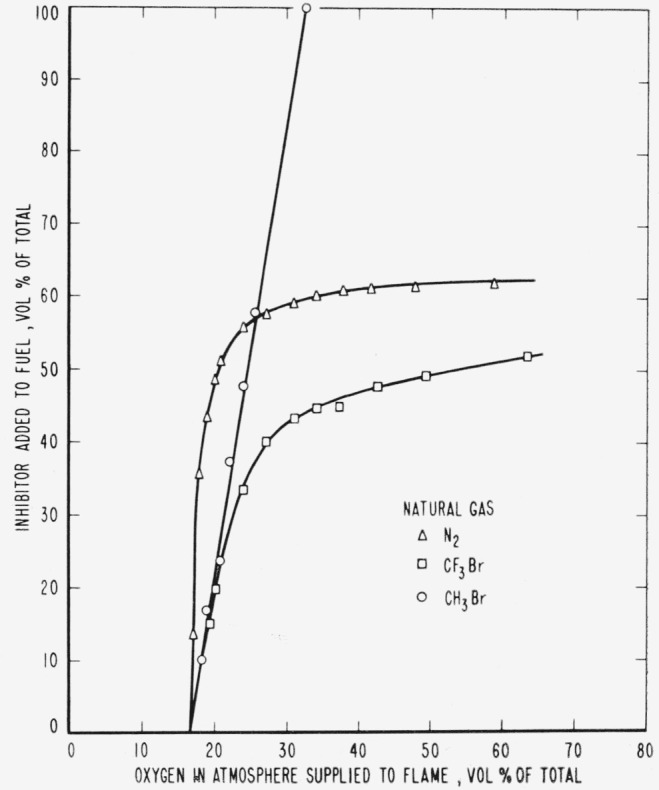
Extinction of natural gas diffusion flames by nitrogen (Δ), *CF_3_Br* (□) and *CH_3_Br* (○) when the inhibitor is added to the fuel.

**Figure 11 f11-jresv65an4p389_a1b:**
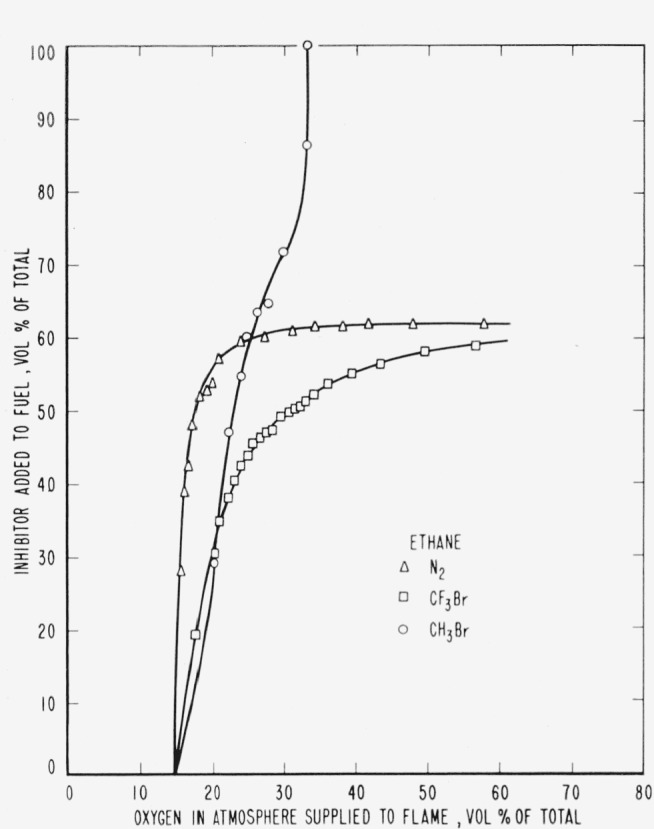
Extinction of ethane diffusion flames by nitrogen (Δ), *CF_3_Br* (□) *CH_3_Br* (○) when the inhibitor is added to the fuel.

**Figure 12 f12-jresv65an4p389_a1b:**
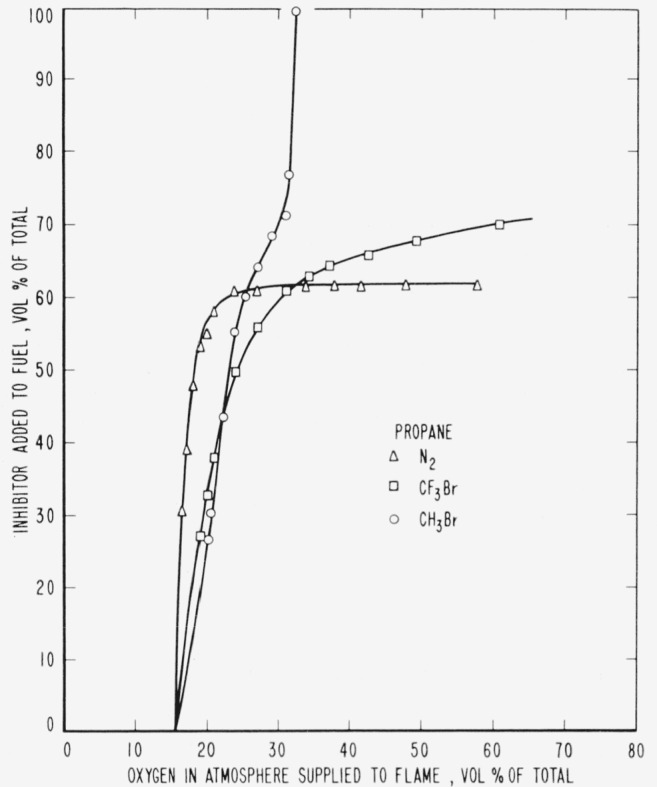
Extinction of propane diffusion flames by nitrogen (Δ), *CF_3_Br* (□) and *CH_3_Br* (○) when the inhibitor is added to the fuel.

**Figure 13 f13-jresv65an4p389_a1b:**
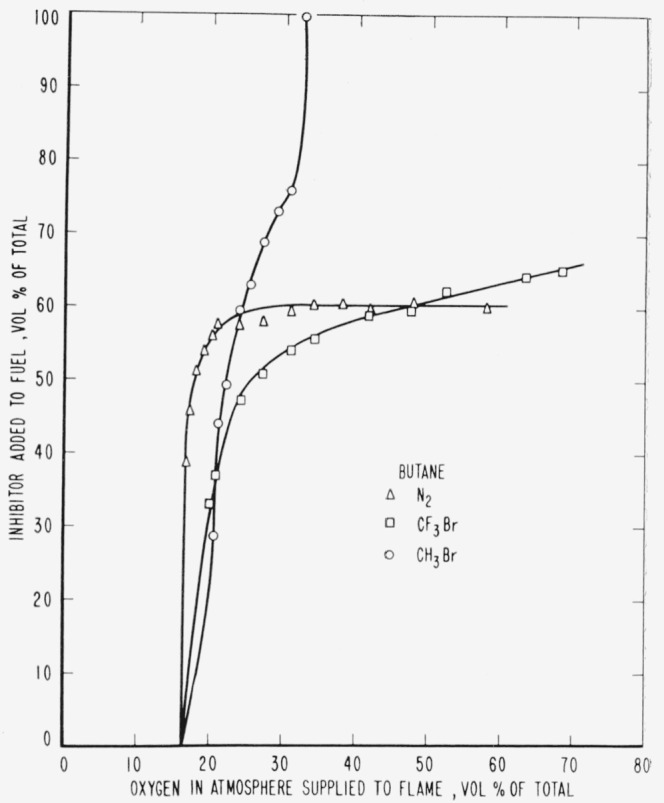
Extinction of butane diffusion flames by nitrogen (Δ), *CF_3_Br* (□) and *CH_3_Br* (○) when the inhibitor is added to the fuel.

**Figure 14:**
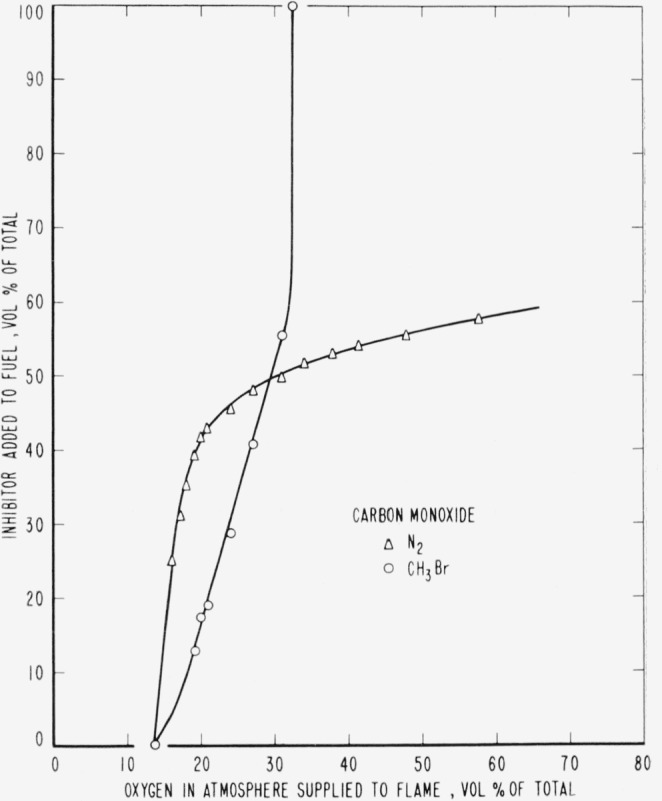
Extinction of carbon monoxide diffusion flames by nitrogen (Δ) and by *CH_3_Br* (○) when the inhibitor is added to the fuel.

The curves of figures 9 to 14, inclusive, show the results of adding methyl bromide, trifluoromethyl bromide or nitrogen to the fuels. As before, the curves are boundaries between regions in which diffusion flames burn or are extinguished, burning taking place under conditions represented to the right and below the curves with extinction above and to the left. [Fig f10-jresv65an4p389_a1b] [Fig f11-jresv65an4p389_a1b] [Fig f12-jresv65an4p389_a1b] [Fig f13-jresv65an4p389_a1b]

No curve is shown for the effect of CF_3_Br added to CO as fuel. The amount required for extinguishment was too small to be measured with the apparatus available. It was estimated, however, to be approximately 1 percent by volume of the fuel rate. It thus appears that the CO diffusion flame was extinguished by a very small amount of CF_3_Br regardless of whether it was added from the oxygen or from the fuel side of the reaction zone. There seemed to be no dependence of extinguishment on the oxygen concentration outside the reaction zone, regardless of whether the inhibitor was added to the fuel or oxygen side of it.

## 4. Discussion

Relative efficiencies of the three inhibitors for various concentrations of oxygen in the atmosphere supplied to the flame may be derived from the curves (fig. 9 to 14, inclusive) for the case where the inhibitor was added to the fuel. However, when the inhibitor was added to the atmosphere surrounding the flame, allowance had to be made for the reduction in oxygen concentration caused by the addition of inhibitor to the starting mixture of oxygen and nitrogen. Lines parallel to the dotted lines for air in figures 3 to 8, inclusive, give the oxygen concentration. Values for the percent inhibitor at extinction when normal air was the starting mixture were read from the curves at points where they crossed the dotted lines and are shown in table 1. Also shown in the table are the efficiencies of the two balogenated inhibitors compared to that of nitrogen. It is evident from the table and curves that, in spite of its inertness, the inhibiting effect of nitrogen is different on opposite sides of the reaction zone. Because of this fact, it was felt that nitrogen provided the best basis for a comparison of the efficiencies of the halogenated compounds when applied to the two sides of the reaction zone. The reaction zone of a diffusion flame may be considered as being bounded on one side by the rich combustible limit and on the other by the lean combustible limit, with the stoichiometric mixture somewhere in between, the whole flame being somewhat diluted by products of combustion and the inerts which accompany the reactants. The results of this study indicate that diffusion flames are peculiar in that the amount of nitrogen they will tolerate is dependent upon whether it appears at the lean or rich boundary. For example the hydrogen diffusion flame will not burn if more than 54 percent nitrogen is present in the fuel, even if the oxygen in the O_2_–N_2_ mixture supplied to the flame is increased to 70 percent (fig. 9). However, if the nitrogen is added with the oxygen, the mixture may contain as much as 94.1 percent nitrogen (fig. 3). In terms of the reaction $H_{2}+\frac{1}{2}\;O_{2}\to H_{2}O$, 6.8 times as much nitrogen may be added with the oxygen as with the fuel. In the absence of chemical effects and neglecting differences in rates of diffusion, it would be expected that the halogenated inhibitors would behave similarly. The results show that, at 21 percent oxygen in the atmosphere supplied to the flame (where it will tolerate the most inhibitor), CF_3_Br gives a value of 0.36 for the ratio of tolerable concentrations on the oxygen and fuel sides of the reaction zone. This value is only 5.4 percent of that for nitrogen, indicating that, while the hydrogen flame behaved similarly toward the addition of nitrogen and CF_3_Br on the fuel side, the effects of these additives were quite different when added on the oxygen side. At lower oxygen concentrations these differences were even more pronounced, as they were for some of the hydrocarbon flames. The effect seems to be absent in CO flames; they behaved differently toward the addition of nitrogen, but CF_3_Br appeared to differ little in its effect whether added to one side or the other.

Figures 3 and 9 and table 1 show that neither of the halogenated inhibitors was particularly effective in extinguishing a hydrogen diffusion flame, either when added to the air side or to the fuel side of the reaction zone. Their effectiveness increased when used on the oxygen side of hydrocarbon flames, CF_3_Br being somewhat more effective than CH_3_Br. However, for the CO diffusion flame there was a great difference not only between the effectiveness of the inhibitors, but also between their effects on the CO flame and the flames of the other fuels. The difference between the effectiveness of the two inhibitors for CO flames is, no doubt, connected with the hydrogen contained in the CH_3_Br molecule and the well-known effects of hydrogen and hydrogen-containing compounds on the combustion of CO. The decreasing effectiveness of both inhibitors as the amount of free and bound hydrogen in the fuel increased suggests that some reaction of hydrogen opposes the action of the inhibitor.

Figures 3 to 14, inclusive, show that the oxygen concentration in the oxygen-nitrogen mixture supplied to the flame has a rather large effect on the efficiency of the inhibitors, particularly at oxygen concentrations below that found in air. The curves for methyl bromide may be interpreted as showing that the inhibitor starts to become a fuel at about 25 percent oxygen concentration and will burn unsupported as a diffusion flame when the oxygen concentration reaches about 32 percent. This result is not unexpected since Marsh [2] gives lower and upper limits of 14 and 19 percent for upward propagation in a 2-in. diameter tube when methyl bromide is mixed with oxygen. Figures 3 to 8, inclusive, show that methyl bromide is not effective as an inhibitor when supplied with an oxygen-nitrogen mixture containing more than 25 percent oxygen. This may be interpreted as evidence that combustion products of methyl bromide are ineffective as inhibitors when added on the oxygen side of the diffusion flame. Trifluoromethyl bromide seems to retain its effectiveness to considerably higher oxygen concentrations. When added to the fuel the inhibitors are most effective at oxygen concentrations below that found in air, but do not approach the corresponding efficiencies for the oxygen side of the reaction zone until the oxygen concentration approaches the minimum required to support the uninhibited flame.

The very low efficiency of the halogenated inhibitors when added to the fuel can probably be attributed to failure of the molecules to survive the combined effects of the reducing atmosphere and pyrolysis to which they are subjected as they enter the reaction zone. The results of this study suggest that there is less chance of survival when the inhibitor is added to the fuel than when added to the oxygen side of the reaction zone.

The lack of effectiveness of the decomposition products of CH_3_Br and CF_3_Br suggest that inhibition may be a result of some reaction or property of the intact inhibitor molecule, or of its freshly released decomposition products. The former concept is not consistent with the theory of Rosser [3] which postulates interference by halogen atoms with chain reactions involving hydrogen atoms. It seems likely that the inhibition reaction takes place in some particular region of the reaction zone, which requires that the inhibitor be stable enough to reach the area where its reaction takes place, but not so stable that it cannot react after it gets there.

The present study with halogenated inhibitors and much other unpublished work in this laboratory has brought to our attention the fact that gases known to capture electrons in other systems (mass spectrometer, discharge tubes, ionization detectors for gas chromatographs [4], etc.) have shown strong influences on the combustion processes when they are added to flames. These gases include oxygen, water vapor, nitrogen dioxide, and halogenated organic compounds. Of the many compounds which show this property, only those which form negative ions having a relatively high electron affinity, such as OH^−^, O^−^ and the negative halogen ions, appear to have important effects. A list of halogenated compounds which are known to capture electrons is given in order of decreasing effectiveness as extinguishers, in table 2 [7]. Compounds in the lower half of the table are rather poor inhibitors and are known to either be strongly bonded or to produce no halogen ions in the mass spectrometer. The parallel between the yield of negative ions [5, 6] by dissociative resonance capture of electrons and the efficiency as a flame inhibitor for a number of these compounds is striking and is under investigation. The resonance dissociative capture of electrons may have something to do with the presence of the red and ultra-violet bromine recombination bands shown in the specta by Simmons and Wolfhard [1].

## 5. Summary

Methyl bromide and trifluoromethyl bromide are much more effective inhibitors when added to the oxygen side of the reaction zone of hydrogen or hydrocarbon diffusion flames than when added to the fuel side. In general, methyl bromide is less effective than trifluoromethyl bromide except at oxygen concentrations below that of air and when added to the fuel. Methyl bromide burns as a diffusion flame, unsupported by additional fuel, at oxygen concentrations above 33 percent. When added to the oxygen side of the reaction zone, it appears to burn as a premixed flame outside the main reaction zone at oxygen concentration above about 25 percent.

For the fuels studied, the difficulty of inhibition increases with the amount of hydrogen in the fuel, when the inhibitor is added to the oxygen side of the reaction zone, but shows no such effect when it is added to the fuel.

The effectiveness of the inhibitors when added to the oxygen side of the reaction zone is a function of the oxygen concentration, methyl bromide being completely ineffective above about 25 percent oxygen, trifluoromethyl bromide retaining some of its effectiveness at much higher oxygen concentrations except for the case of hydrogen as the fuel.

The results have practical application in that they show that the inhibitors are more effective under conditions of low oxygen concentrations and when mixed with the air supplied to a fire.

## Figures and Tables

**Table 1:** Comparison of extinguishment characteristics of *N_2_*, *CH_3_Br*, and *CF_3_Br* for various fuels burning in air

Fuel	Percentage of inhibitor in air or fuel at extinction						Efficiency relative to nitrogen			
	When added to air			When added to fuel			Added to air		Added to fuel	
	N_2_	CH_3_Br	CF_3_Br	N_2_	CH_3_Br	CF_3_Br	CH_3_Br	CF_3_Br	CH_3_Br	CF_3_Br
										
H_2_	94.1	11.7	17.7	52.4	58.1	52.6	8.0	5.3	0.9	1. 0
CH_4_	83.1	2.5	1.5	51.0	28.1	22.9	33.2	55.4	1.8	2.2
C_2_H_6_	85.6	4.0	3.0	57.3	36.6	35.1	21.4	28.5	1.6	1.6
C_3_H_8_	83.7	3.1	2.7	58.3	34.0	37.6	27.0	31.0	1.7	1.6
C_4_H_10_	83.7	2.8	2.4	56.8	40.0	37.9	29.9	34.9	1.4	1.5
CO	90	7.2	0 8	42.8	19.9	….	12.5	112	2.2	….

**Table 2:** Qualitative parallelism between extinguishing efficiency and dissociative resonance capture of electrons

Compound	Concentration for extinguishment at peak flammability [7]	Electron attachment	Halogen ion formed	Reference
				
CH_3_I	6.1	yes	yes	6
CBrF_3_	6.1	yes	yes	5
CF_3_I	6.8	yes	yes	5, 11
CHBrF_2_	8.4	yes	yes	9
CH_3_Br	9.7	yes	yes	6
CClF_2_ CClF_2_	10.8	yes	yes	8
CCl_4_	11.5	yes	yes	9, 8, 11
CClF_3_	12.3	yes	yes	5
CF_3_CF_3_	13.4	yes	yes	9
CCl_2_F_2_	14.9	yes	yes	8, 11
C_4_F_8_	18.1	yes	yes	9
SF_6_	20.5	yes	no	8
HCl	25.5	yes	yes	11
CF_4_	26.0	yes	yes	5, 8
CH_3_Cl	…	no	no	6
CH_3_F	…	no	no	6
C_8_F_16_O	>8^a^	yes	no	10

a Unpublished work from this laboratory.

## Footnotes

1 Figures in brackets indicate the literature references at the end of this paper.

2 Analyses furnished by the supplier.
